# Decision making under uncertainty, therapeutic inertia, and physicians’ risk preferences in the management of multiple sclerosis (DIScUTIR MS)

**DOI:** 10.1186/s12883-016-0577-4

**Published:** 2016-05-04

**Authors:** Gustavo Saposnik, Angel Perez Sempere, Roula Raptis, Daniel Prefasi, Daniel Selchen, Jorge Maurino

**Affiliations:** Division of Neurology, Department of Medicine, St. Michael’s Hospital, University of Toronto, 55 Queen St E, Toronto, ON M5C 1R6 Canada; Neuroeconomics and Decision Neuroscience, Department of Economics, University of Zurich, Zurich, Switzerland; Department of Neurology, Hospital General Universitario de Alicante, Alicante, Spain; Applied Health Research Center, Li Ka Shing Knowledge Institute, St. Michael’s Hospital, University of Toronto, Toronto, Canada; Neuroscience Area, Medical Department, Roche Farma, Madrid, Spain

## Abstract

**Background:**

The management of multiple sclerosis (MS) is rapidly changing by the introduction of new and more effective disease-modifying agents. The importance of risk stratification was confirmed by results on disease progression predicted by different risk score systems. Despite these advances, we know very little about medical decisions under uncertainty in the management of MS. The goal of this study is to i) identify whether overconfidence, tolerance to risk/uncertainty, herding influence medical decisions, and ii) to evaluate the frequency of therapeutic inertia (defined as lack of treatment initiation or intensification in patients not at goals of care) and its predisposing factors in the management of MS.

**Methods/Design:**

This is a prospective study comprising a combination of case-vignettes and surveys and experiments from Neuroeconomics/behavioral economics to identify cognitive distortions associated with medical decisions and therapeutic inertia. Participants include MS fellows and MS experts from across Spain. Each participant will receive an individual link using Qualtrics platform^©^ that includes 20 case-vignettes, 3 surveys, and 4 behavioral experiments. The total time for completing the study is approximately 30–35 min. Case vignettes were selected to be representative of common clinical encounters in MS practice. Surveys and experiments include standardized test to measure overconfidence, aversion to risk and ambiguity, herding (following colleague’s suggestions even when not supported by the evidence), physicians’ reactions to uncertainty, and questions from the Socio-Economic Panel Study (SOEP) related to risk preferences in different domains. By applying three different MS score criteria (modified Rio, EMA, Prosperini’s scheme) we take into account physicians’ differences in escalating therapy when evaluating medical decisions across case-vignettes.

**Conclusions:**

The present study applies an innovative approach by combining tools to assess medical decisions with experiments from Neuroeconomics that applies to common scenarios in MS care. Our results will help advance the field by providing a better understanding on the influence of cognitive factors (e.g., overconfidence, aversion to risk and uncertainty, herding) on medical decisions and therapeutic inertia in the management of MS which could lead to better outcomes.

**Electronic supplementary material:**

The online version of this article (doi:10.1186/s12883-016-0577-4) contains supplementary material, which is available to authorized users.

## Background

The field of multiple sclerosis (MS) has seen significant changes over the last several years [1, 2]. Clinicians and patients welcomed the introduction of disease-modifying therapy (DMT) for MS in the mid-1990s. Injectable agents, all with rather similar risk–benefit profiles, dominated MS care for over a decade. The approval of Natalizumab marked a change with the introduction of a more effective treatment option, but also entailed new risks associated with modulation of the immune system (e.g., risk of progressive multifocal leukoencephalopathy - PML) [2, 3]. More recently, the introduction of oral agents and new humanised monoclonal antibodies administered by infusion have opened yet another avenue for patients and clinicians [4]. Currently, there are over a dozen of DMTs available to treat MS, with varying availability around the world. Significant heterogeneity exists in the efficacy and risks associated with these therapies [5–7]. Therefore, clinicians have the challenge of tailoring treatment based on i) disease activity level (clinical and radiological data), ii) individual patient characteristics/preferences, iii) personal expertise/preference, in order to identify the optimal balance between efficacy and safety Table 1 (See Additional file 1 for data on some currently available agents) [8].

**Table 1:** Comparative adverse events of different DMTs [7, 8]

Disease modifying agent	Adverse events
Interferon beta	• Depression
	• thrombotic microangiopathy
	• hepatotoxicity
	• ISRs
	• Flu-like
	• LFT elevation
	• Leukopenia
Glatiramer acetate	• ISRs
	• Benign systemic reaction
Mitoxantrone	• Cardiac toxicity
	• Leukemia
Natalizumab	• Infusion reactions
	• PML
	• Infusion-related fatigue
Fingolimod	• Bradyarrhythmia
	• Macular edema
	• Herpes virus infection
	• PML
	• BCC
	• LFT elevation
	• Lymphopenia
	• Mild hypertension
Teriflunomide	• Hepatotoxicity
	• Peripheral neuropathy
	• Alopecia
	• Nausea/Diarrhea
Dimethyl fumarate	• Flushing
	• Gastrointestinal
	• PML
Alemtuzumab	• Infusion reactions
	• ITP
	• Goodpasture syndrome
	• Thyroid cancer
	• Infections
	• Autoimmune thyroid disease

*ISRs* injection-site reactions, *LFT* liver function test, *PML* progressive multifocal leukoencephalopathy, *ITP* idiopathic thrombocytopenic purpura, *BCC* basal cell carcinoma

### Risk stratification in MS

**Fig. 1:**
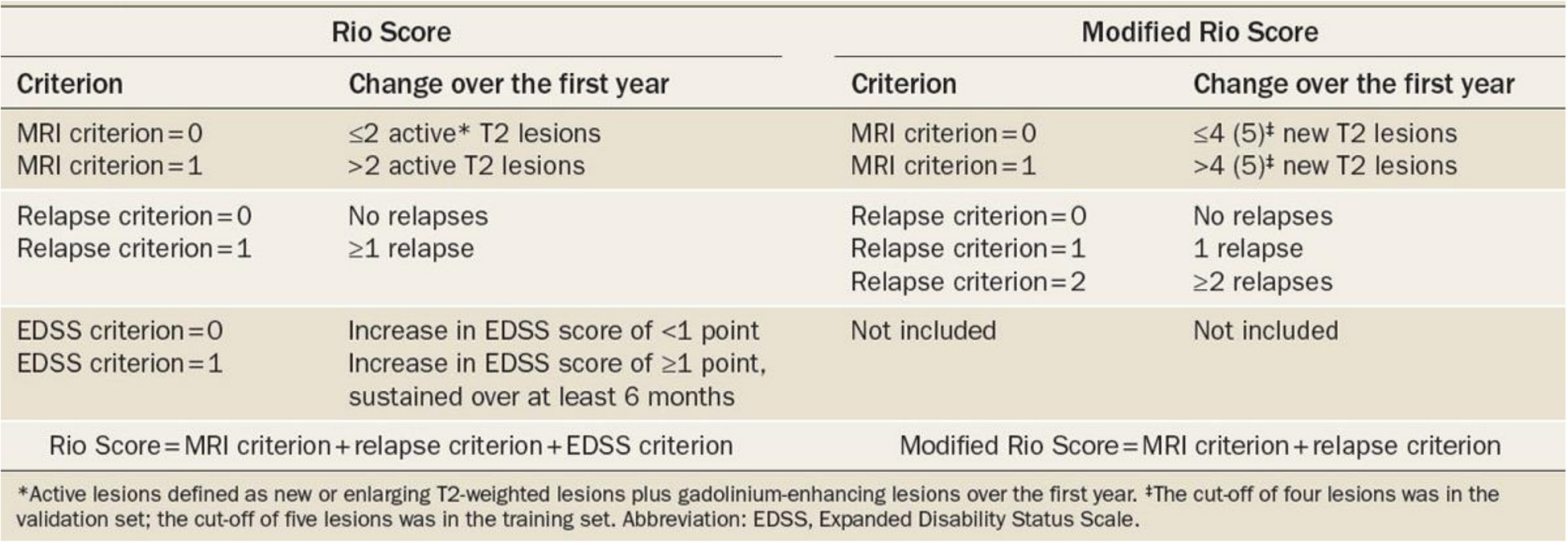
Modified with permission from Sormani et al. defining and scoring response to IFN‑β in multiple sclerosis. Nat. Rev. Neurol. doi:10.1038/nrneurol.2013.146

An understanding of the risk of untreated multiple sclerosis is crucial to make therapeutic decisions Table 2 [8]. In addition, physicians’ preferences and beliefs in effectiveness of treatment and drug safety profiles may influence their decisions. Disease activity/progression can be divided into physical, cognitive and radiological markers. Examples include number of attacks per year, number of disabling attacks, disability scales (clinical), lesion volume, GAD enhancing lesions, brain atrophy (MRI), and cognitive decline (e.g., using SDMT, PASAT, OR MoCA scales) [9]. Two scoring systems (Rio score and Modified Rio score) demonstrate good predictive value for MS progression. The Rio score includes MRI, clinical relapse and EDSS criteria, whereas the modified Rio score includes MRI and clinical relapse criteria (Fig. 1) [10]. A high risk profile using the modified Rio (score ≥2) includes either an MRI with more than 5 new T2 lesions (1 point) or 1 relapse in the first year (1 point) or two relapses within the first year of treatment (2 points) or the combination of these criterions [11]. These scores have been used to identify and predict response to Interferon β. For example, the modified Rio score in the PRISM trial revealed that participants who did not responded to Interferon β had a similar probability of disability progression as those assigned to the placebo group. Conversely, responders to Interferon β had a 52 % reduction in disability progression compared to placebo and non-responders (*p* < 0.0001). MS patients with a modified Rio score greater than or equal to 2 had a 65 % increased risk of disability progression (HR = 4.60; *p* < 0.001) [12]. A Canadian group concluded that a change in treatment may be considered in patients with relapsing remitting MS if there is a high level of concern in any one domain (relapses, progression or MRI), a medium level of concern in any two domains, or a low level of concern in all three domains [13]. The European Medicines Agency approves escalating therapy with Natalizumab or Fingolimod in patients who had at least one relapse in the previous year while on Interferon β and either ≥9 T2-hyperintense lesions on brain MRI or ≥1 contrast-enhancing lesion MRI activity alone after the first year of treatment was associated with three- to fivefold increased risk of relapses or disability compared with stable patients. These recommendations have been supported by several prospective studies [14, 15].

**Table 2:** Risks of untreated relapsing MS

Treatment targets	Evidence of association	Long-term outcome
T2 lesion volume	Increase of 0.8–l ml/year	Correlates with increased relapse frequency and long term disability outcomes.
T1 black hole conversion	40–50 % of lesions go on to form black holes	Correlation with clinical measures and disability progression.
Brain atrophy	0.5–1 %/year in MS vs. <0.1 % in healthy controls	Correlation with cognitive outcomes and EDSS in the long term.
Clinical relapses	Annualized relapse rate in placebo arms: 0.5–1.38	Relapses associated with decreased quality of life.
		Relapses associated with accrual of disability.
		Earlier onset of SPMS.
Disability accrual	Average change of 0.27 EDSS points/per relapse	Increased likelihood of long term disability.
	MRI and lesional activity associated with disability progression	

Reproduced with permission from Ontaneda et al. [8]

Selection of a first line therapy will likely depend on several factors. Traditionally, and due to the availability of extended safety data, injectable agents may be the first choices. Given the comparable efficacy data between the injectable agents the selection of a therapy will be determined mostly by side effect profiles. Subjects with headaches, depression, and a history of liver dysfunction may experience worsening of these comorbidities when exposed to interferons. Monitoring for interferons includes following liver function tests, complete blood counts, and monitoring depression [8]. Given the availability of more effective drugs, the treatment paradigm is likely to change. However, it is expected there will be wide variability on the timing of this paradigm shift (e.g., starting more effective therapies as first line treatment) based on patients’ and physicians’ tolerance to risk, estimation of the clinical course, regional funding programs, among other factors. As a result, it is vital to identify situations for which physicians take the opportunity of escalating treatment when indicated (e.g., progression of disease determined by clinical relapses, EDSS disability score and imaging data).

### Therapeutic inertia: a new paradigm in MS

Therapeutic inertia is a term introduced in 2006 to define the lack of treatment initiation or intensification in patients not at goals of care [16–19]. Some examples include failure to intensify treatment in patients with persistent elevated blood pressure or blood glucose [16, 20, 21]. Reasons to explain therapeutic inertia include the lack of training and cultural organization in the practice at “treating to target”, competing demands and clinical uncertainty [22, 23]. In the context of MS, therapeutic inertia is defined lack of treatment initiation or intensification when there is evidence of disease progression (based on clinical and radiological data). In the present study, disease progression was defined according to the modified Rio score, where patients had one or more recurrent attacks and/or an MRI with 5 or more new T2 lesions while receiving treatment with a disease- modifying agent [11]. Another more recent criterion strongly associated with risk of relapse or disability progression was the presence of isolated gadolinium-enhancing lesions [14, 15].

## Medical decision making

Making decisions in medical care is a complex task involving a variety of cognitive processes [24]. Decision making is defined as the process of examining possibilities, risks, uncertainties, and options, comparing them, and choosing a course of action [25, 26]. Decisions based on erroneous assessments may result in incorrect patient and family expectations, and potentially suboptimal advice, treatment, and prognosis. Moreover, many decisions are made with limited information from observational studies or clinical trials that may not apply to particular patients. Uncertainty is one of the most important reasons contributing to the status quo and making proactive therapeutic decisions [17, 23, 27]. We need a better understanding on how physicians decide about different therapeutic options under uncertainty for patients with MS.

## The problem

Despite the availability of different markers for risk stratification in patients with MS, it is difficult for expert clinicians to select the best strategy when the progression pattern of the disease is uncertain. MS experts and clinicians are trained to quickly recognize patterns or critical aspects of particular situations [28]. Some clinicians apply the knowledge they have acquired from previous experience, others use information available at the time of the assessment, others use risk score tools or a combination of the above. However, it is not known how MS experts behave in clinical scenarios with ambiguous outcomes (unknown probability or uncertain risk of an outcome) or when more therapeutic options become available. In addition, we have a limited understanding about physicians’ beliefs and preferences on the widely available therapeutic options for the optimal management of MS.

Moreover, there is still lack of evidence-based approaches to incorporate patients’ preferences such as medication disutility into the shared decision making process [29]. As our understanding of MS risk continues to be refined, how to account for the uncertain risks, benefits, and preferences at the individual level is a current challenge for the practice of personalized medicine.

## The proposed solution: bringing together advances in MS treatment and Neuroeconomics

**Fig. 2:**
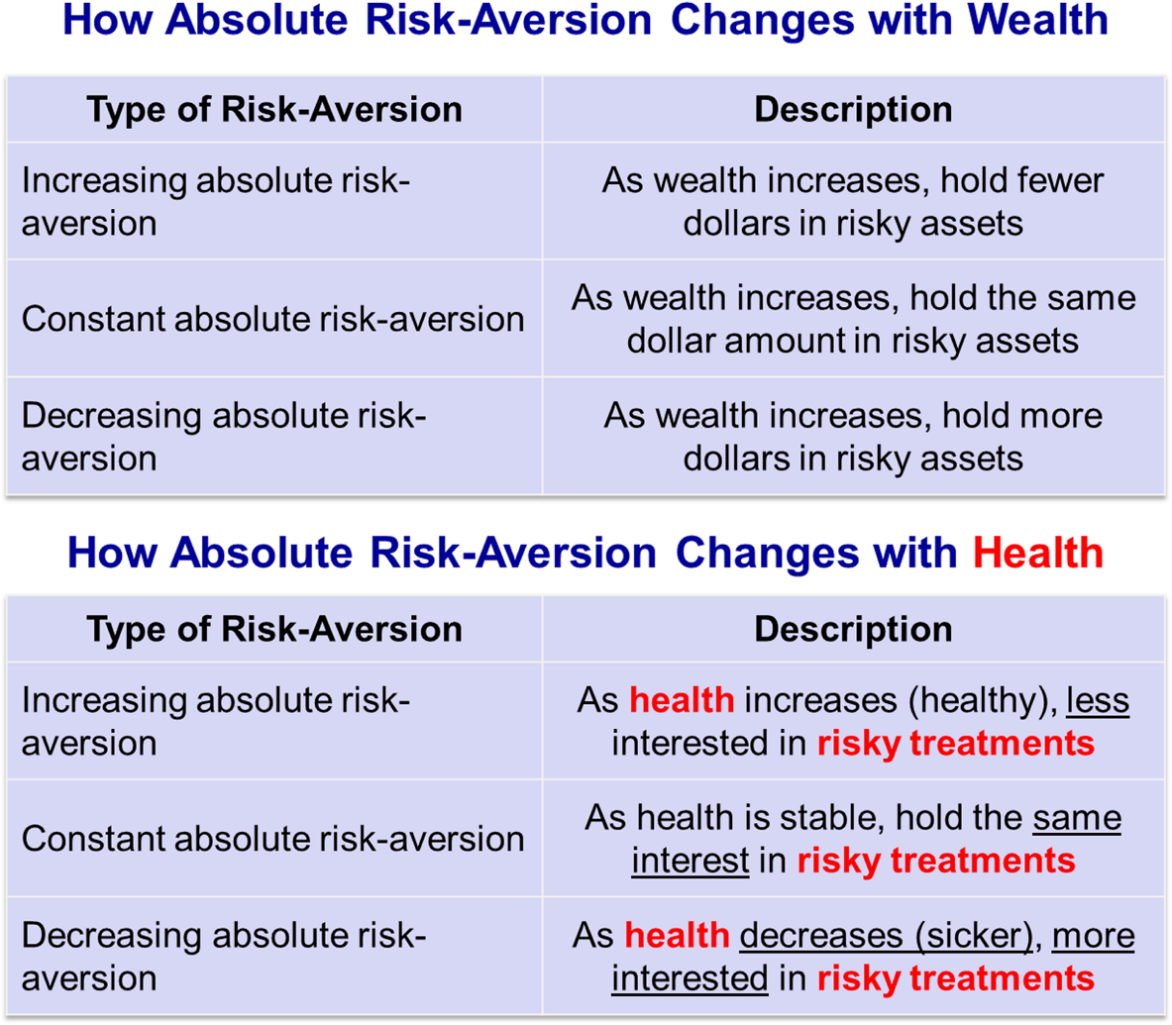
Illustrative comparison of risk aversion changes as a function of wealth and health

The expected utility theory states that decision makers choose between risky or uncertain options by comparing their expected utility values (i.e.,: the weighted sums obtained by adding the utility values of outcomes multiplied by their respective probabilities) [30]. More importantly, patients’ preferences and physicians’ recommendations will change depending on the utility function of their current health status. For example, patients at low risk of developing MS progression may prefer to avoid ‘risky’ treatments (as they have low gains while having a risk of developing side effects), whereas high-risk patients would prefer the most effective treatment even if need to take higher risks (as they have a higher chance of having a progression leading to more disability) (Fig. 2) [24, 31].

## Rationale

Neuroeconomics is the science that studies the principles of how we make decisions [30, 32]. The neuroscience of decision making is based on behavioral economic concepts and mathematical approaches, such as game theory, to predict and model how people make their own choices [33].

**Fig. 3:**
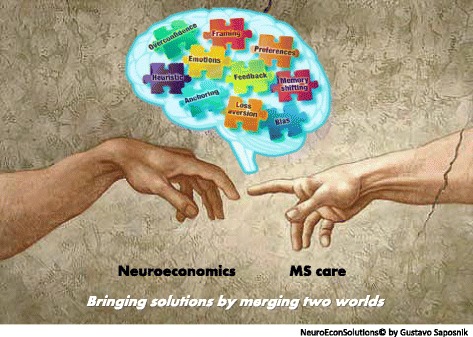
Framework

The application of principles from Neuroeconomics (decision neuroscience) will facilitate the recognition of physicians’ therapeutic preferences and beliefs about DMT for MS in the real world [34] (Fig. 3). Given the greater availability of treatment options, MS treatment will likely become more challenging. It requires a fine balance between the modest benefits of the less expensive, safer, and traditional DMTs versus new agents, usually more costly with potential harmful side effects. The so called ‘intermediate agents’ (e.g., Fingolimod) may have a ‘decoy effect’ (Phenomenon whereby consumers tend to have a specific change in preference between two options when also presented with a third -less preferable- option becomes available) [35, 36].

There is limited evidence of the application of the expected utility theory to clinical scenarios from the physicians’ perspective. A better understanding of physicians’ beliefs and preferences under uncertainty would likely improve the quality of care, patients’ satisfaction, and likely improve clinical outcomes by increasing awareness on the therapeutic inertia in MS.

## Objectives

1. To evaluate tolerance to risk and ambiguity among MS experts under situations of uncertainty.
2. To assess the prevalence of ‘therapeutic inertia’.
3. To determine the influence of tolerance to risk/ambiguity, overconfidence and herding on medical decisions.

## Research questions

1. How MS experts’ perceptions of risk and tolerance to ambiguity influence their recommendations?
2. What is the prevalence of therapeutic inertia among physicians with expertise in MS?
3. What is the impact of tolerance to risk and ambiguity, overconfidence and herding on therapeutic decisions?

## Methods

We are proposing a prospective web-based study comprising 20 MS case-vignettes, 3 standardized surveys, and 4 behavioral experiments (see Additional file 1).

MS case-scenarios were derived from the most common situations in clinical practice as identified by experts in the field. Surveys include three standardized questions related to aversion to risk from The German Socio-Economic Panel (SOEP) study. The SOEP is a longitudinal study of private households that include household composition, occupational biographies, employment, earnings, health and satisfaction indicators [37, 38]. The English version is available online [39].

Based on our previous work including a systematic literature review of studies evaluating cognitive biases or distortions in the medical field, we selected tolerance to risk and ambiguity, overconfidence, herding, and decisions about someone else [40]. We used the physician’s reaction to uncertainty test to assess tolerance to risk or ambiguity in patient care [41]. This questionnaire comprised an initial pool of 61 items [41]. Factor analysis of the 428 respondents revealed a high accuracy (Cronbach's alpha = 0.90). The short version of this questionnaire includes five questions [42].

Behavioral experiments were designed to elicit risk and ambiguity aversion in the health and financial domains [43, 44], herding (decisions influenced by other colleagues) [45], decisions about someone else vs. own, and overconfidence (perception that own judgments are more accurate or in the top 50 % of the studied population) [46].

### Participants

Neurologists actively involved in the care of patients with MS from across Spain will be invited to participate in our study. Invitations are facilitated by the Spanish Society of Neurology (Sociedad Española de Neurologia). We use Qualtrics platform for the design and implementation of our study. It is expected physicians will be able to complete the study within 30 min.

Participating physicians will receive fair market compensation for the time involved in completing the survey.

### Outcome measures

The primary outcome of the study is the proportion of participants who exhibit aversion to ambiguity and therapeutic inertia [19, 43]. Ambiguity aversion is defined as is a preference for known risks over unknown risks [43]. This can be elicited through the experiments #16 and #17 in the health and financial domains (Additional file 1).

Therapeutic inertia will be assessed based on the selected treatment options in case-scenarios with recurrent relapses, appearance of new brain lesions in follow up MRI’s while taking a disease modifying agent over a specified period. Secondary outcome measures include the association between risk aversion, overconfidence, and herding with therapeutic decisions and the assessment of therapeutic inertia using different criteria.

#### Sample size calculation

Based on the results of pilot studies evaluating other medical conditions (e.g., atrial fibrillation) and our systematic review on the frequency of cognitive distortions affecting physicians [40], we require a sample size of 120 physicians (60 per group) (Table 3) to reach 90 % power to detect a conservative 20 % absolute difference in therapeutic inertia between participants exposed and not exposed to cognitive distortions.

**Table 3:** Sample size calculation

Power^a^	90 %	85 %	80 %
N (per group)	60	53	46

^a^The power was calculated to detect a 20 % absolute difference between groups (40 % vs 20 %) with an alpha of 5 % (two-sided) for all of the calculations in the table

#### Feasibility

the study interventions are simple and doable. The protocol includes clinical scenarios commonly observed in clinical practice. According to the Spanish Neurological Society (Sociedad Española de Neurología-SEN), there are over 1600 neurologists, 13 specialized MS centers comprising approximately 200 specialists in the field in Spain. Assuming a low response rate of 50 %, the completion of our study is feasible considering the required sample size to reach a power of 90 % with an alpha of 5 %.

### Analytical plan

To address objective 1, we will characterize participants’ risk and ambiguity aversion as identified by the corresponding experiments (Additional file 1, behavioral battery questions (Q) #1 to 4.

To address objective 2, we evaluate therapeutic inertia (TI) as elicited by 10 case-vignettes. We will created a TI score representing the number of cases that participants did not escalate treatment (numerator) over 10 (denominator) multiplied by 100. The diversity of case-scenarios will also allow the analysis of therapeutic inertia using different criteria (e.g., modified Rio score, European Medicines Agency, isolated GAD-enhanced lesions).

To address objective 3, we will complete a univariate and multivariable analysis to determine the influence of risk aversion, tolerance to ambiguity, overconfidence and herding on therapeutic decisions and TI score.

Chi squared tests will be used to compare categorical variables; *t*-test or Kruskal-Wallis tests will be used to compare mean and median differences for continuous variables. The primary analysis will evaluate the association between physicians’ responses in the behavioral component of the survey with responses in the case-scenarios. A multivariable analysis will be completed to determine the association between physicians’ characteristics with the primary outcome of interest. Adjustment includes the following variables: age, sex, years of experience, expertise, volume of MS patients seen per week, and practice setting (academic vs. community). All tests were 2-tailed, and p-values <0.05 will be considered significant.

#### Knowledge translation strategies

We plan to take a multifaceted approach to knowledge translation, targeting the following audiences for communication: 1) Neurologist, 2) the clinical academic community, 3) the media, 4) policy-makers, and 5) MS patients and their families. We expect to generate high impact publications and media interest to inform the public and influence MS care programs. This work is also expected to increase awareness about therapeutic inertia among MS experts and to contribute toward new guidelines for the management of MS. We are working with key stakeholders to discuss the most effective dissemination strategy and target the key messages for all audiences.

## Discussion

Patients and physicians caring for patients with MS are confronted with important uncertainties concerning the diagnosis, prognosis, disease course, and disease-modifying therapies. In the recent years, new therapeutic alternatives became available for management of MS [5, 47]. These advances were achieved by targeting different pathophysiological mechanisms, producing more effective DMTs, but accompanied by either higher risk of infections, or more serious side effects [48]. As a result, MS experts have an expanded therapeutic arsenal compared to a decade ago. Decisions are not merely about the selection of an injectable interferon or Glatiramer (given daily or every other day) usually accompanied by skin reactions or flu-like symptoms, but rather the individual selection of the most appropriate DMT (e.g., dose, administration type, efficacy and safety profile) according to disease severity, patient’s clinical status and preference. Consequently, more effective agents are now more accessible for MS patients who failed traditional DMT [5, 49].

Interestingly, physicians have limited education in both risk management and in formal training in decision making [50, 51].

We are proposing a novel approach in expanding research of MS care by combining case-vignettes with the assessment of cognitive distortions through experiments in Neuroeconomics (Decision Neuroscience). The application of Neuroeconomics’ principles may help overcome those barriers by identifying and increasing awareness about cognitive distortions (e.g., overconfidence, tolerance to risk and ambiguity, etc.) that may lead to suboptimal decisions (e.g., therapeutic inertia) [18, 25, 52].

This study will provide evidence about: i) how MS experts make decisions under uncertainty, ii) how MS experts would change their preferences based on their tolerance to risk and ambiguity, iii) the prevalence of therapeutic inertia based on different criteria for escalating therapy (modified Rio, European Medicines Agency), and iv) the influence of cognitive distortions on therapeutic inertia.

DIScUTIR MS is designed as a pilot study to determine the feasibility of assessing tolerance to risk and ambiguity, therapeutic inertia, and associated factors among practicing physicians with expertise in MS.

The results of our study will also facilitate crucial information to understand current MS care practices and how physicians’ preferences (e.g., risk aversion) have a global impact on medical and daily life decisions.

Some limitations need to be acknowledged. First, the small sample size of MS experts from a single country (Spain) would limit the generalizability of the results. However, DIScUTIR MS is designed as a pilot study to determine the feasibility of a larger worldwide study. Second, the concept and definition of therapeutic inertia applied to MS care is not widely disseminated. Some colleagues may also argue about the absence of an accepted definition of therapeutic inertia in MS care. However, we used a widely acceptable definition of TI supported by studies showing health care improvements in the management of key and widely prevalent conditions (i.e.,: blood pressure and diabetes).

Despite the aforementioned limitations, our study will increase physicians’ awareness of crucial situations under uncertainty in the management of MS. The results of DIScUTIR MS will provide a starting point to ignite discussions about a widely accepted definition of therapeutic inertia in MS care. This is relevant considering the lack of MS guidelines concerning clinical scenarios under uncertainty or progression of disease [53, 54].

The identification of clinical or radiological progression in MS should at least set the time of ‘therapeutic momentum’ to consider escalating treatment, especially when cost-effective options are available. In this setting, physicians may want to take that opportunity to discuss risk-benefit scenarios in a way similar to how financial advisors assess their clients’ preferences and risk tolerance when advising about a variety of investment portfolios. An open discussion in risky situations following the appropriate documentation of disease progression would ameliorate the therapeutic inertia and may lead to more optimal decisions in the care of patients with MS.

### Ethics approval

The protocol was approved by the Research Ethics Board of St. Michael’s Hospital, University of Toronto. Consent will be obtained by agreeing to participate in the study.

### Availability of data and materials

The appendix contains all details of the protocol.

## Additional file

Additional file 1: Case-vignettes and behavioral experiments. (DOCX 601 kb)

## Abbreviations

DMT: disease modifying agents
EMA: European Medicines Agency
MS: multiple sclerosis
SOEP: Socio-Economic Panel Study
