# Supplementary material for: Decision making under uncertainty, therapeutic inertia, and physicians’ risk preferences in the management of multiple sclerosis (DIScUTIR MS)
Source: BMC Neurol. 2016 May 4;16:58. doi: 10.1186/s12883-016-0577-4 (PMC4855476; doi:10.1186/s12883-016-0577-4)
Supplement: Additional file 1: — Case-vignettes and behavioral experiments. (DOCX 601 kb) [file 12883_2016_577_MOESM1_ESM.docx]

Additional file 1

**DEMOGRAPHICS**

| Current age (years): ________  Sex: O Female  O Male  Specialty:  General Neurologist  Neurologist specialized in MS  Other (specify): _________  ______________________  Average number of MS patients seen per week:______  Have you attended the ECTRIMS this year? Yes NO | Practice setting (please check one):  O Academic  O Community  O Both (Academic and Community)  O Other (specify): _______________________________________  Percentage of time you spend in clinical practice  (please check one): O 0-25%  O 26-50%  O 51-75%  O > 75%  Total number of years in practice: ___________  In the last 3 years, have you been a co-author of a scientific  Publication in a peer-reviewed journal? Yes No |
| --- | --- |

**PART 1 - BEHAVIORAL**

| **1. The earth’s equator is around … (please check the one correct option).**  O 40, 000 km long  O 24, 000 km long  O 36, 000 km long  O 52, 000 km long  O 14, 000 km long |
| --- |

| \| **2. The solar system consists of … known planets (please check the one correct option).**  O 13  O 81  O 12  O 91  O 17 \| \| --- \| |
| --- | --- |

| **3. The first Tour de France took place in the year … (please check the one correct option).**  O 1898  O 1915  O 1903  O 1814  O 1938 |
| --- |

| \| **4. Which triplet of musical notes comprises C major chord (please check the one correct option).**  O A-C-B  O D-F-A1  O C-D-G1  O F-C-D1  O C-E-G \| \| --- \| |
| --- | --- |

| \| **5. Ludwig van Beethoven wrote … symphonies (please check the one correct option).**  O 151  O 911  O 410  O 130  O 104 \| \| --- \| |
| --- | --- |

| \| **6. The electric frequency in central Europe is … (please check the one correct option).**  O 220 Hz  O 110 Hz  O 50 Hz  O 66 Hz1  O 85 Hz \| \| --- \| |
| --- | --- |
| \| **7. The human cell consists of … chromosomes (please check the one correct option).**  O 32  O 581  O 46  O 381  O 23 \| \| --- \| |

| \| **8. The human body has … sense organs (please check the one correct option).**  O 4  O 51  O 6  O 71  O 8 \| \| --- \| |
| --- | --- |
| \| **9. The sum of all angles in a triangle is … (please check the one correct option).**  O 360 degrees  O 380 degrees1  O 60 degrees  O 90 degrees1  O 180 degrees \| \| --- \| |
| \| **10. The “Lord of the Rings” is a book by … (please check the one correct option).**  O Tolkien  O Tolstoy1  O Trotzki  O Thomas1  O Trevier \| \| --- \| |
|  |
|  |
| \| **11.** For the last 10 questions that you just answered above **(questions #1 through 10)**, on a scale of 0 to 10, how confident do you  feel about your responses?  **0 1 2 3 4 5 6 7 8 9 10**  **Not confident**  **Confident** \| \| --- \| \| **12.** For the last 10 questions that you just answered above **(questions #1 through 10)**, how many questions do you think you  answered correctly?  **Number of questions answered correctly:**  **0 1 2 3 4 5 6 7 8 9 10** \| \| **13.** For the last 10 questions that you just answered above **(questions #1 through 10)**, on a scale of 0 to 10, how would you rate  your responses compared to your peers?  **0 1 2 3 4 5 6 7 8 9 10**  **Above the top 50%**  **Below the top 50%** \| |

**14.** Imagine you are given two options to receive a monetary prize:

Option 1 (50/50): A 50/50 chance of winning $400 or $0

Option 2 (Safe): A sure, secured amount of money

What is the minimum amount of money you would accept from the safe option instead of the 50/50 option? Please check one.


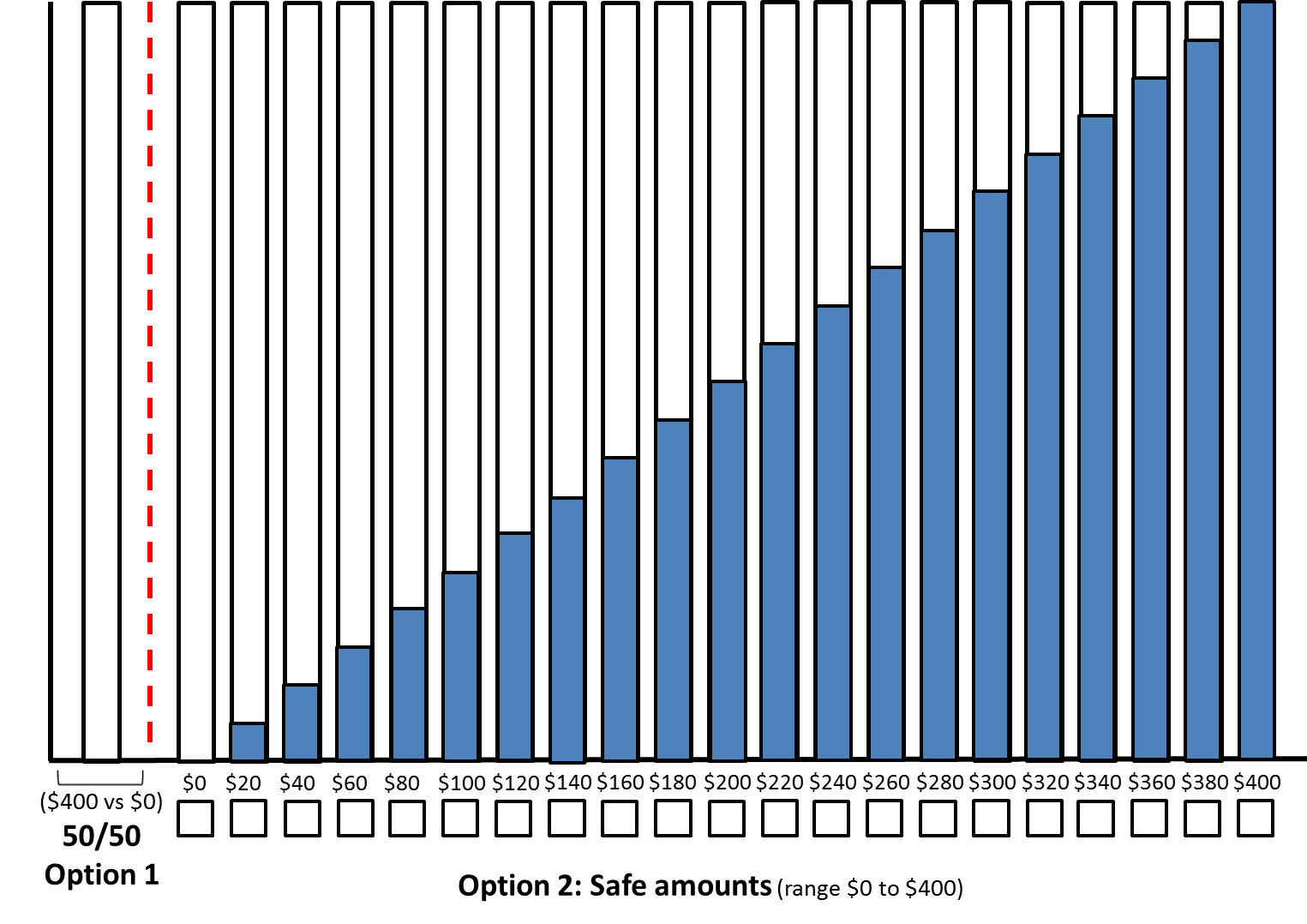


**15.** You are seeing a patient in your clinic and there are two treatment options.

Option 1: The treatment will result in:

-survival of 20 years;

-20% chance of serious side effects, including potential hospitalization and disability.

Option 2: No treatment, which has lower survival.

What is the minimum **healthy years of survival** with **no treatment** (and therefore, **no side effects**) that you would be comfortable

with? Please check one.


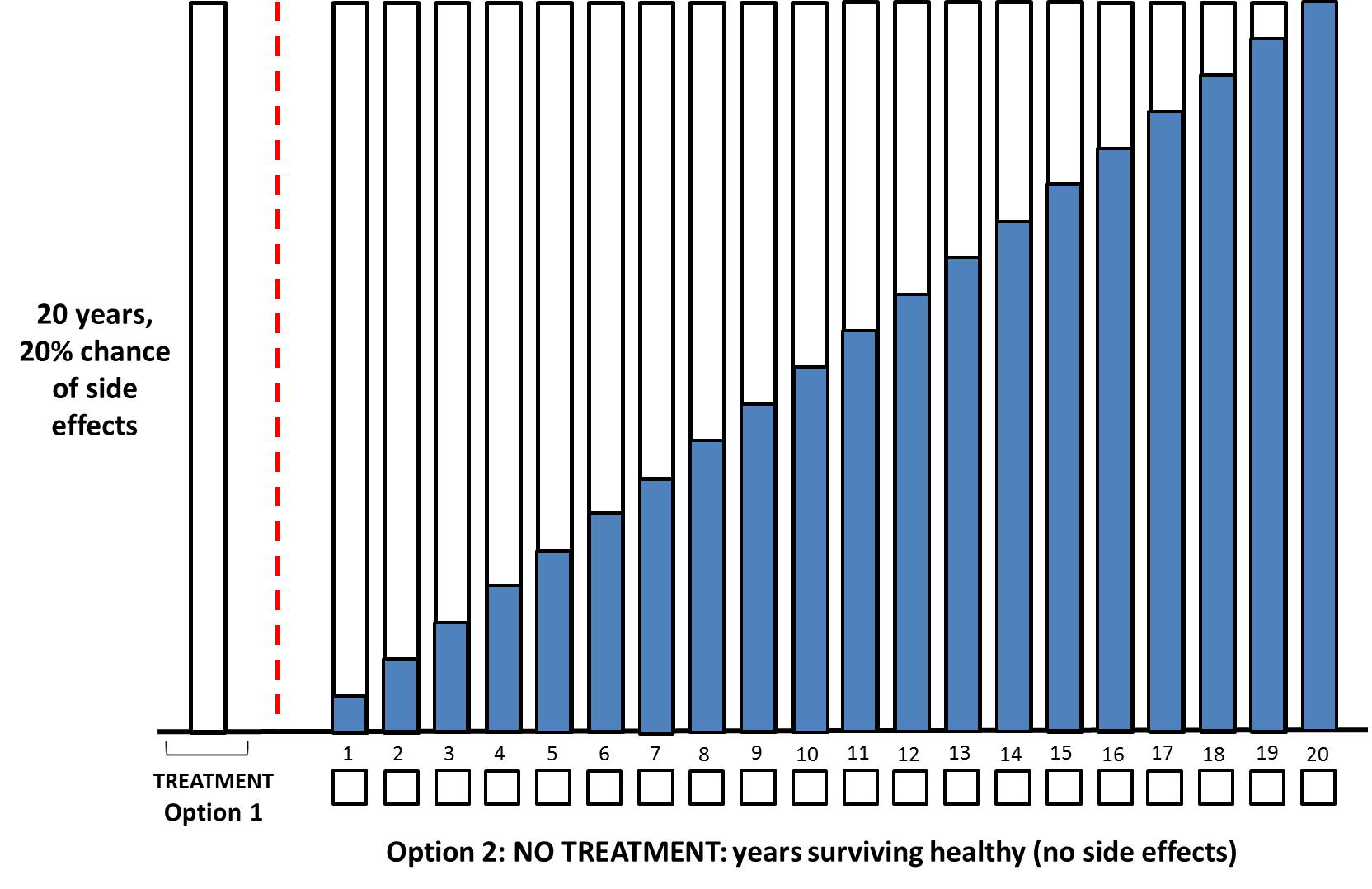


**16.** Suppose you have two different types or urns

Urn A: you know that 50% of the balls are red and the other 50% are blue.

Urn B: you do not know the exact proportion of blue to red balls.

If you draw a **blue ball**, **you win the full amount of $400**. If you draw a red ball, you win $0.

For each of the following scenarios (1 to 9), which urn would you select? Please check one – either Urn A (50/50 option) ***or*** Urn B

(with the grey bar representing the unknown proportion of balls) for each of the 9 scenarios.

**For example:** for scenario 1, you have to mark the checkbox next to Urn A (50/50 chance of winning $400) or the one next to Urn

B (grey bar representing the unknown proportion of balls) according to your preference. Similar for the remaining scenarios.


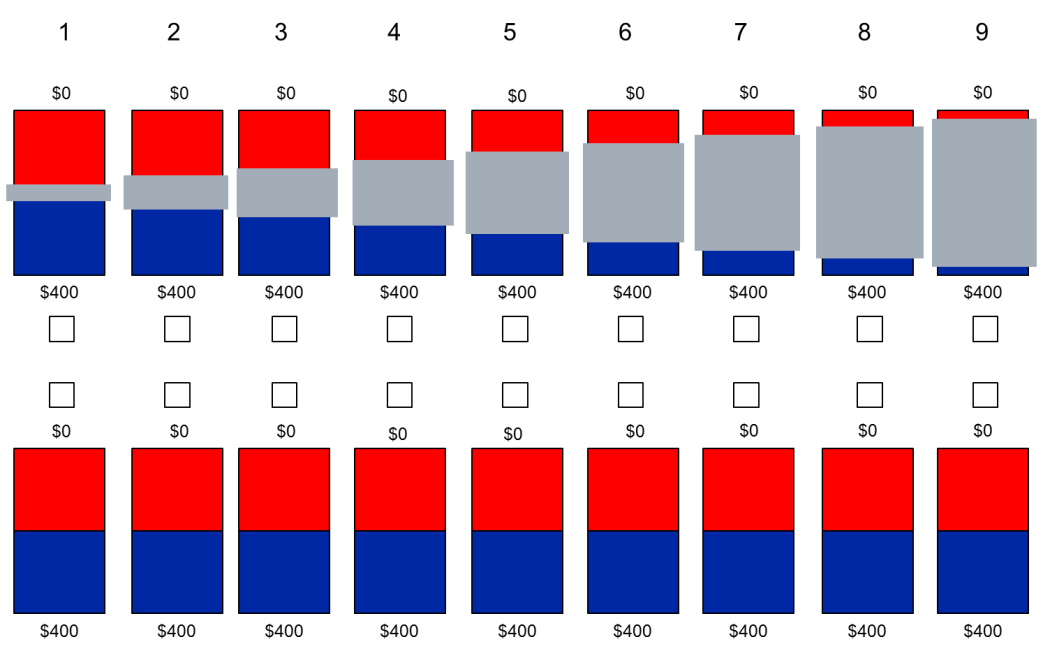


**Urn B**

**Urn A**

**Scenario**

**17.** You are seeing a patient who developed a medical condition for which there are two effective treatments available, “Treatment A” and “Treatment B”

With “Treatment A”, the patient will have 50% probability of survival.

With “Treatment B”, the patient will have some probability of survival, but you do not know the exact probability.

For EACH of the following scenarios (1 to 9), which treatment would you choose?

Please check one – either “Treatment A” (50% probability of survival) ***or*** “Treatment B” (unknown probability of survival with

the grey bars representing how much is unknown about the probability of survival) according to your preference.

| **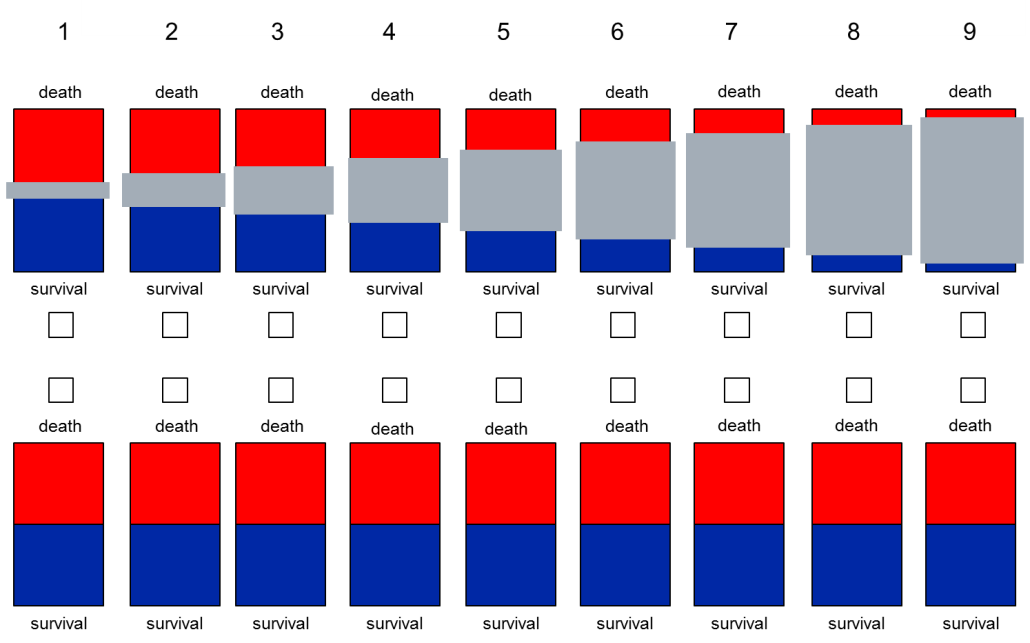**  **Treatment B**  Probability of survival is unknown  **Treatment A**  50% probability of  survival  **Scenario** |  |  |  |
| --- | --- | --- | --- |
| **18. How would you rate your willingness to take risks in the following areas?**  **Please answer on a scale from 0 (not at all) to 10 (very much).** | | |  |
| 1. **While driving.**   **0 1 2 3 4 5 6 7 8 9 10**  **Not at all**  **Very much** | | |  |
| 1. **In financial matters.**     **0 1 2 3 4 5 6 7 8 9 10**  **Strongly Disagree**  **Strongly Agree** | | |  |
| 1. **During leisure and sports.**     **0 1 2 3 4 5 6 7 8 9 10**  **Strongly Disagree**  **Strongly Agree** | | |  |
| 1. **In your occupation.**     **0 1 2 3 4 5 6 7 8 9 10**  **Strongly Disagree**  **Strongly Agree** | | |  |
| 1. **With your health.**     **0 1 2 3 4 5 6 7 8 9 10**  **Strongly Disagree**  **Strongly Agree** | | |  |
| 1. **Your faith in other people.**     **0 1 2 3 4 5 6 7 8 9 10**  **Strongly Disagree**  **Strongly Agree** | | |  |
| **19. On a scale from 0 to 10, what is your willingness to accept moderate side effects if the treatment is effective?**  **0 1 2 3 4 5 6 7 8 9 10**  **Always willing to take risks**  **Always avoid risks** | | | |

| **20. When you think about the future, are you … (please check one)**  **0 1 2 3 4 5 6 7 8 9 10**  **Pessimistic Optimistic** |
| --- |

**21.** You will be asked to decide whether an urn contains the majority of red or blue marbles.

There is a 50% chance that the urn contains two red marbles and one blue marble, and a 50% chance the urn contains two blue marbles and one red marble.

The first participant draws a marble from the urn; he looks at the color and then places it back in the urn without showing it to the rest of the class.

Then, he/she announces his guess: majority-red or majority-blue. Each participant is rewarded if his/her guess was correct.

Participant one: announces red-majority

Participant two: announces red-majority

**Now, it is your turn.** You draw one blue marble.

What is your guess for the urn? Please select one:

O Red-majority

O Blue majority

| 1. **On a scale from 0 to 100, what is the maximum probability of moderate side effects that you are willing to accept for an effective treatment of a CHRONIC condition (e.g. MS, PD)?**     Please, write a number : **_____ (from 0 to 100)** |
| --- |

| 1. **On a scale from 0 to 100, what is the maximum probability of moderate side effects that you consider acceptable for an effective treatment of an ACUTE condition (e.g. MS flare, stroke)?**   Please, write a number: **_____ (from 0 to 100)** |  |
| --- | --- |
| **PHYSICIANS REACTION TO UNCERTAINTY**  **24. On a scale from 0 to 10, please express your level of agreement with the following statements:** | |
| 1. **The uncertainty of patient care often troubles me.**   **0 1 2 3 4 5 6 7 8 9 10**  **Strongly Disagree**  **Strongly Agree** | |
| 1. **I find the uncertainty involved in patient care disconcerting.**     **0 1 2 3 4 5 6 7 8 9 10**  **Strongly Disagree**  **Strongly Agree** | |
| 1. **I usually feel anxious when I am not sure of the diagnosis.**   **0 1 2 3 4 5 6 7 8 9 10**  **Strongly Disagree**  **Strongly Agree** | |
| 1. **Uncertainty in patient care makes me uneasy.**     **0 1 2 3 4 5 6 7 8 9 10**  **Strongly Disagree**  **Strongly Agree** | |
| 1. **I am quite comfortable with the uncertainty in patient care.**     **0 1 2 3 4 5 6 7 8 9 10**  **Strongly Disagree**  **Strongly Agree** | |

**PART 2 – CASE-VIGNETTES**

1. A 42-year old woman diagnosed of RRMS 8 years ago has been under IM interferon (IFN) beta1a treatment.

Her last relapse was 3 years ago. She complaints of memory difficulties and lack of attention in the last 12 months. Her husband confirms the cognitive symptoms affecting her daily activities. No evidence of a mood disorder. Symbol Digit Modalities Test (SDMT) score of 43 (a score of 55 or lower accurately classified cognitive impairment in MS patients).

Current brain MRI: 2 new T2 periventricular lesions, no T1 Gd-enhanced lesions.

**i) What would you do? Please select one:**

. Continue on the same DMT and perform a new neuropsychological evaluation in 6 months

. Stop IFN and start her on a monoclonal antibody agent (natalizumab/alemtuzumab)

. Stop IFN and start her on Fingolimod

. Stop IFN and start her on Glatiramer acetate

. Stop IFN and start her on Dimethyl fumarate

**ii) How confident are you with your decision?**

**0 1 2 3 4 5 6 7 8 9 10**

**Very confident**

**Not confident**

1. A 23-year old woman with a diagnosis of RRMS in treatment with SC interferon beta1a since the last year. EDSS score of 2.0.

A recent brain MRI performed one year after the initiation of IFN showed 6 new T2 bilateral periventricular lesions. There was no T1 Gd-enhanced lesions.

**Would you continue on the same DMT?**

. Yes

. No

**If you chose No, What would you do? Please select one:**

. Stop IFN and start her on monoclonal antibody (natalizumab/alemtuzumab)

. Stop IFN and start her on Fingolimod

. Stop IFN and start her on Dimethyl fumarate

. Stop IFN and start her on Glatiramer acetate

1. A 32-year old woman with a diagnosis of RRMS in treatment with Dimethyl fumarate since the last 18 months. Baseline MRI showed six T2 periventricular and one brainstem hyperintense lesions.

Clinically stable. EDSS score of 1.0. During treatment the levels of lymphocytes have been low for more than 6 months (between 0.2–0.5×10^9^/L)

**i) What would you do? Please select one:**

. Continue on Dimethyl fumarate and perform a new lymphocyte count in 3 months

. Stop Dimethyl fumarate and start her on an Interferon beta or Glatiramer acetate

. Stop Dimethyl fumarate and start her on Fingolimod

. Stop Dimethyl fumarate and start her on Teriflunomide

. Stop treatment until lymphopenia resolution and then restart Dimethyl fumarate

. Stop Dimethyl fumarate and start her on a monoclonal antibody (natalizumab/alemtuzumab)

**ii) How confident are you with your decision?**

**0 1 2 3 4 5 6 7 8 9 10**

**Very confident**

**Not confident**

1. A 27-year old woman with a diagnosis of RRMS has been on SC interferon beta1a (IFN) for 18 months. She had two recurrent events since the initiation of IFN. Her EDSS score is 2.0.

A control brain MRI revealed 6 new T2 bilateral periventricular lesions and one subcortical Gd-enhanced lesion compared to the MRI prior to the initiation of IFN:

**What would you do? Please select one:**

. Stop IFN and start her on Teriflunomide

. Stop IFN and start her on Fingolimod.

. Stop IFN and start her on Ocrelizumab.

1. A 25-year old man, previously healthy, developed pain and blurred vision on his right eye 1 month before. An ophthalmologist confirmed the diagnosis of optic neuritis. A brain MRI showed nine T2 periventricular and one pontine hyperintense lesions, and one T1 Gd-enhanced juxtacortical lesion .

**What would you do? Please select one.**

- Start treatment with an injectable DMT (INF beta or Glatiramer acetate)
- Start treatment with Fingolimod
- Start treatment with Ocrelizumab
- Start treatment with Dimethyl fumarate
- Start treatment with Alemtuzumab
- Start treatment with Teriflunomide
- Start treatment with Natalizumab

1. A 38-year old woman diagnosed with RRMS 12 years ago. She has been taken Fingolimod for 3 years. Clinically and radiological stable.

She presents to your office for a follow-up visit. Her white blood count revealed a lymphopenia < 0,2x10^9^/l.

**i) What would you do? Please select one:**

. Stop treatment until lymphopenia resolution

. Continue on fingolimod because the lymphopenia threshold under Fingolimod is < 0,1x10^9^/l.

. Stop Fingolimod and start her on an injectable DMT (INF beta or Glatiramer acetate)

. Stop Fingolimod and start her on Dimethyl fumarate

. Stop Fingolimod and start her on a monoclonal antibody (natalizumab/alemtuzumab)

**ii) How confident are you with your decision?**

**0 1 2 3 4 5 6 7 8 9 10**

**Very confident**

**Not confident**

1. A 29-year old woman with a diagnosis of RRMS has been on SC interferon beta1a (IFN) for 16 months. She had two recurrent events since the initiation of IFN. Her EDSS score is 2.0.

A control brain MRI revealed 6 new T2 bilateral periventricular lesions and one subcortical Gd-enhanced lesion compared to the MRI prior to the initiation of IFN:

**What would you do? Please select one:**

. Stop IFN and start her on Teriflunomide.

. Stop IFN and start her on Fingolimod.

. Stop IFN and start her on Natalizumab

. Stop IFN and start her on Ocrelizumab

1. A 40-year old man diagnosed of RRMS 3 years ago (EDSS score of 2) developed a new onset of imbalance and double vision. He had 2 previous relapses (optic neuritis and hypoesthesia in right upper limb). He has been on SC interferon beta 1a since the diagnosis. A new brain MRI showed two T1 Gd-enhanced lesions involving the brainstem and upper cerebellar peduncle.

**i) What would you do? Please select one**

- Stop IFN and start him on Glatiramer acetate
- Continue on the current treatment
- Stop IFN and start him on a monoclonal antibody (natalizumab/alemtuzumab)
- Stop IFN and start him on Teriflunomide
- Stop IFN and start him on Fingolimod
- Stop IFN and start him on Dimethyl fumarate

**ii) How confident are you with your decision?**

**0 1 2 3 4 5 6 7 8 9 10**

**Very confident**

**Not confident**

1. A 37-year old woman with a diagnosis of RRMS 5 years ago went to your clinic for a follow-up. In the past, she had 3 relapses while treated with IM interferon once weekly. Then, she was switched to Fingolimod being clinically stable during the last 2 years. Her EDSS score is 4. Clinically stable. A new brain MRI revealed a new T1 Gd-enhanced periventricular lesion.

**What would you do? Please select one**

- Continue with the current therapy and repeat an MRI in 6 months
- Stop Fingolimod and start her on Dimethyl fumarate
- Stop Fingolimod and start her on Teriflunomide
- Stop Fingolimod and start her on a monoclonal antibody agent (natalizumab/alemtuzumab)

1. A 50-year old woman diagnosed of MS 15 years before, showed a continuous disability progression during the last 8 months not associated to a relapse. She had a total of 6 relapses while being treated with IM IFN once weekly, Glatiramer acetate, and Fingolimod, which it is her current therapy. EDSS score: 5.5. A brain MRI showed three T2 new periventricular hyperintense lesions and two T1 new “black holes”.

**i) What would you do? Please select one.**

- Stop all treatments
- Stop Fingolimod and start her on sc INF beta 1a because she has a SPMS
- Stop Fingolimod and start her on a monoclonal antibody (natalizumab/alemtuzumab)
- Stop Fingolimod and start her on sc INF beta 1a
- Stop Fingolimod and start her on Dimethyl fumarate
- Continue with the current therapy

**ii) How confident are you with your decision?**

**0 1 2 3 4 5 6 7 8 9 10**

**Very confident**

**Not confident**

1. A 32-year old woman diagnosed of RRMS 2 years ago came to your office for a follow-up appointment. She had 2 previous relapses. She has been receiving Dimethyl fumarate since the diagnosis. Clinically stable.

A follow-up brain MRI showed three new T2 hyperintense lesions, no T1 Gd-enhanced lesions.

**What would you do? Please select one.**

- Continue on the current therapy
- Stop DMF and start her on Teriflunomide
- Stop DMF and start her on Fingolimod
- Stop DMF and start her on a monoclonal antibody agent (natalizumab/alemtuzumab)

1. A 29-year old man diagnosed with MS was started treatment with Dimethyl fumarate. EDSS score of 2.5. Clinically stable.

He noticed nausea, abdominal pain and diarrhea since he started Dimethyl fumarate one year ago.

**What would you do? Please select one.**

- Continue with Dimethyl fumarate and say him that gastrointestinal side effects will improve in a longer period
- Stop Dimethyl fumarate and start him on fingolimod
- Stop Dimethyl fumarate and start him on teriflunomide
- Continue with Dimethyl fumarate at half dose
- Stop Dimethyl fumarate and start him on IFN/glatiramer acetate
- Stop Dimethyl fumarate and start him on a monoclonal antibody agent (natalizumab/alemtuzumab)

1. A 25-years old woman developed a facial palsy and headache. Neurological examination showed a right peripheral facial palsy and hypoesthesia on the left upper limb. A MRI showed a T1 Gd-enhancing lesion in the right-pons and multiple T2 periventricular and juxtacortical hyperintense lesions. You treated her with methylprednisolone and then started Glatiramer acetate treatment. Six months later, she developed a new relapse with loss of strength in the right arm.

**Would you continue on the same DMT?**

. Yes

. No

**If you chose No, What would you do? Please select one:**

**What would you do? Please select one.**

- Stop GA and start her on Fingolimod
- Stop GA and start her on an IFN beta
- Stop GA and start her on Dimethyl fumarate
- Stop GA and start her on a monoclonal antibody agent (natalizumab/alemtuzumab)
- Stop GA and start her on Teriflunomide

1. A 47-year old male was diagnosed with RRMS 10 years ago. He has a new relapse with loss of strength on his right leg. An MRI revealed a new Gd enhancing lesion on left frontal lobe. His baseline EDSS was 6 and he needed a cane for walking. Following this new relapse he is unable to walk, and required a wheelchair. He has been treated with Fingolimod for the last 3 years. Previously received Glatiramer acetate and SC IFN every other day.

**What would you do? Please select one**

- Continue the treatment with Fingolimod
- Stop Fingolimod and start him on Natalizumab
- Stop Fingolimod and start him on Terflunomide
- Stop Fingolimod and start him on Alentuzumab
- Stop Fingolimod and start him on Dimethyl fumarate
- Stop any treatment

1. (to determine if follows other colleague recommendation of not changing when appropriate to change for another DMT)-

A 35 yo woman was diagnosed with MS 5 years ago. She has been stable for the last 3 years on Glatiramer. Two month ago, she developed a left sided weakness and double vision lasting 3 weeks. An MRI of the brain revealed 4 GAD enhancing lesions (2 periventricular and 2 subcortical 15 mm in size and on in the right tegmentum of the pons).

She was seen by an MS colleague in your absence who recommended continuing on Glatiramer. Her EDSS is currently 2.5, whereas one year ago was 1.5.

She came back to your office to get your opinion.

What would you recommend? **Select only one**

- Stop Glatiramer and start her on a new disease modifying agent
- Continue on Glatiramer as recommended

1. A 45 year old man was diagnosed with MS 10 years ago. He had 3 neurological events in total. MRI of the brain revealed 5 periventricular/pericallosal lesions. He has been stable on an interferon agent during this time. He heard about new oral agents and would like to switch for an oral compound.

**i) What would you do? Please select one.**

O Stop the IFN agent and start him on Teriflunomide

O Stop the IFN agent and start him on Dimethyl Fumarate

O Stop the IFN agent and start him on Fingolimod

O Continue on the current IFN beta

O Offer another DMT agent with a different profile (e.g. natalizumab/alemtuzumab)

1. A 39 year old woman was diagnosed with MS 7 years ago. She had 5 neurological events in total. The MRI of the brain revealed confluent periventricular lesions, 5 yuxtacortical lesions and a T4-6 lesions. There is a single periventricular GAD-enhanced lesion (1 cm). She has been stable on Glatiramer acetate during this time. She heard about new oral agents and would like to switch for an oral compound.

**i) What would you do? Please select one.**

O Stop the Glatiramer and start her on Teriflunomide

O Stop the Glatiramer and start her on Dimethyl Fumarate

O Stop the Glatiramer and start her on Fingolimod

O Continue on Glatiramer

O Offer another DMT agent with a different profile (e.g. natalizumab/alemtuzumab)

1. A 54 year old man presented to your office with a one-year history of progressive leg weakness. He denied any previous neurological events. There is no history of recent vaccinations, exposure to substances, fever or constitutional symptoms. His neurological examination revealed spastic paraparesis with brisk reflexes in the lower extremities.

Routine blood work is normal. Chest X-ray is normal. An MRI of the brain revealed 7 periventricular lesions and a T4 hyperintensity of 2 cm.

**i) How confident are you about the diagnosis of Primary progressive MS?**

**0 1 2 3 4 5 6 7 8 9 10**

**Very confident**

**Not confident**

**ii) How would you rate your assessment compared to your peers (expert in MS)?**

**0 1 2 3 4 5 6 7 8 9 10**

**Better
(top 50%)**

**Worse
(low 50%)**

1. A 23 year old woman has been recently diagnosed with MS with 8 yuxtacortical lesions (1 cm each) and 5 Gd- enhancing periventricular lesions (largest 3 cm).
2. **On a scale from 0 to 10, how likely are you to recommend starting Natalizumab?**

**0 1 2 3 4 5 6 7 8 9 10**

**Very likely**

**Not likely**

**ii) If YOU were in a similar situation, how likely is it that YOU would start Alemtuzumab?**

**0 1 2 3 4 5 6 7 8 9 10**

**Very likely**

**Not likely**

**iii) On a scale from 0 to 10, how likely would you start her on disease modifying therapy?**

**0 1 2 3 4 5 6 7 8 9 10**

**Very likely**

**Not at all**

**iv) In the same patient, how do you value an oral agent (vs. an inyectable):**

**0 1 2 3 4 5 6 7 8 9 10**

**More preferable**

**Less preferable**

**v) How preferable is an agent that requires two IV infusions per year (each lasting 4 hours) compared to subcutaneous weekly injections?**

**0 1 2 3 4 5 6 7 8 9 10**

**Not very**

**Very much**

**vi) How do preferable is an agent that requires two IV infusions per year (each lasting 4 hours) compared to daily oral agents?**

**0 1 2 3 4 5 6 7 8 9 10**

**Not very**

**Very much**

1. (to determine if follows other colleague recommendation of changing for another DMT when NOT appropriate to change.

A 40 yo woman was diagnosed with MS 3 years ago. She has been taking IFN SC with no significant side effects. Three month ago, she developed bilateral leg weakness and urinary urgency, which resolved within 2-3 weeks. An MRI of the brain at that time revealed a total of 10 T2 periventricular and yuxtacortical lesions, similar to her baseline MRI. There were no GAD enhancing lesions. Her neurological examination is unchanged from last year, as well as, her EDSS of 1.5.

She expressed some concerns about her recent symptoms while being on DMTs. She was seen by an MS colleague in your absence who recommended switching to Fingolimod.

She came back to your office to get your opinion.

What would you recommend? **Select only one**

- Support starting her on Fingolimod as recommended
- Continue on IFN SC.

**21.** In a scale from 0 to 10, How do you rate the overall performance of EACH agent (performance takes into account the overall efficacy AND safety profile)

[NOTE: use ONE SCALE or slider for each agent]

**List of agents:**

- IFN beta 1a (Avonex/Rebif)
- Alemtuzumab (Lemtrada)
- Glatiramer (Copaxone)
- Fingolimoid (Gilenya)
- Dimetyl Fumarate (Tecfidera)
- Teriflonomide (Aubagio)
- Natalizumab (Tysabri)
- Peginterferon beta 1a (Plegridy)
- Ocrelizumab

**0 1 2 3 4 5 6 7 8 9 10**

**Excellent**

**performance**

**Poor performance**

**THANK YOU FOR YOUR PARTICIPATION**
